# National Trends of Antiparkinsonism Treatment in Taiwan: 2004–2011

**DOI:** 10.1155/2016/1859321

**Published:** 2016-02-18

**Authors:** Weng-Ming Liu, Ruey-Meei Wu, Chia-Hsuin Chang, Jou-Wei Lin, Ying-Chun Liu, Chin-Hsien Lin

**Affiliations:** ^1^Department of Neurology, Buddhist Tzu Chi General Hospital, Hualien, Taiwan; ^2^Department of Neurology, National Taiwan University Hospital, No. 7, Chunag-Shan South Road, Taipei 100, Taiwan; ^3^Department of Medicine, College of Medicine, National Taiwan University, Taipei, Taiwan; ^4^Institute of Preventive Medicine, College of Public Health, National Taiwan University, Taipei, Taiwan; ^5^Cardiovascular Center, National Taiwan University Hospital, Yun-Lin Branch, Douliou, Yunlin County, Taiwan

## Abstract

*Background*. Several guidelines for Parkinson's disease (PD) management were recently updated. We examined temporal trends for antiparkinsonism drugs in Taiwan.* Methods*. Antiparkinsonism prescriptions, including levodopa, ergot/nonergot dopamine agonists (DAs), amantadine, selegiline, entacapone, and anticholinergics, were identified in the Taiwan National Health Insurance Database from 2004 to 2011. Time trend analyses were estimated assuming Poisson distribution.* Results*. A total of 19,302 PD patients in 2004 and 41,606 PD patients in 2011 were analyzed. Antiparkinsonism prescriptions increased significantly from 187,137 in 2004 to 414,587 in 2011. Levodopa monotherapy or combination therapy was the mainstay. Levodopa monotherapy comprised 37.4% of prescriptions in 2004 and 44.2% in 2011, with an annual increase rate of 18.14%. There was a substantially increasing trend of DA prescriptions, which were higher in younger-aged patients (<60 years) than in older-aged group (*p* = 0.0006). Among combination therapy, DA combined with levodopa or other antiparkinsonism medications became the main combinations for younger-aged patients after 2009. After 2005, the proportion of ergot DA usage markedly decreased and PD patients using nonergot DA increased.* Conclusions*. Levodopa was the major treatment from 2004 to 2011. There was a steeply increased trend of DA use, especially in younger-aged patients. Nonergot agents comprised the major DA group after 2005.

## 1. Introduction

Parkinson's disease (PD) is the second most common neurodegenerative disorder, with symptoms of tremor, rigidity, bradykinesia, and postural instability. The prevalence of PD is predicted to double by 2030 [1]. In Taiwan, the prevalence of PD has gradually increased to 147 per 100,000 and the incidence was 28.8 per 100,000 in 2011 [2]. The mainstay of treatments for PD is limited to supplemental therapy including oral treatment with levodopa, dopamine agonists (DAs), monoamine oxidase-B inhibitors (MAOBI), and catechol-O-methyltransferase inhibitors [3]. Previous studies suggested that delayed use of levodopa and administration of DAs may decrease the risk of levodopa-related motor complications [4, 5], although one long-term follow-up study in United Kingdom showed similar risks of motor complications between levodopa and DA after a 14-year follow-up period [6]. This debate raised the recently updated consensus for the management of PD in which DA and MAOBI are suggested as initial treatment for younger-aged, early-stage PD patients (aged < 60 years) and levodopa remains the primary option for older-aged patients with PD (aged ≥ 60 years) [7].

Previous studies have indicated a correlation between ergot DA, including cabergoline, pergolide, and bromocriptine, and a risk of restrictive cardiac valvulopathy [8, 9]. Since then, pergolide was voluntarily withdrawn from the United States market in 2007. Ergot DA has been substantially replaced by nonergot DA, encompassing pramipexole, ropinirole, and rotigotine [10]. However, it is now widely recognized that neuropsychiatric side effects, especially impulse control disorder, are a major limitation to nonergot DA therapy, which has a major effect on the choice of these drugs as initial therapy in younger-aged patients [11].

Since prescribing trends for antiparkinsonism medications are influenced by development of new drugs and changes in knowledge about efficacy and side effects, few have focused on the utilization trends with either monotherapy or combination therapy in PD management [12–14]. The Taiwanese government launched the National Health Insurance (NHI) program in March 1995, which covered more than 99% of the total population by the end of 2008 [15]. It offers comprehensive health insurance to all citizens. Therefore, we examined the patterns and temporal trends of antiparkinsonism drug use from 2004 to 2011, with particular emphasis on different age groups of patients with PD.

## 2. Methods

### 2.1. Data Source

The NHI Research Database was developed at the National Health Research Institute, with linked data from the demographic and enrollment records, hospital claims, ambulatory care visits, and pharmacy-dispensing claims from hospitals, outpatient clinics, and community pharmacies. Every individual in Taiwan has a unique personal identification number. The data on patient identities are scrambled cryptographically by the National Health Research Institutes to protect patient's privacy.

Our study was approved by the Research Ethics Committee of National Taiwan University Hospital. No informed consent from participants was required because the data were analyzed anonymously.

### 2.2. Study Population

We searched the Taiwan National Health Insurance Database for the entire population from 2004 to 2011. Patients were included as (1) diagnosed with PD (International Classification of Diseases, 9th Revision, Clinical Modification, ICD-9-CM code: 332.0) and (2) diagnosed with at least one PD diagnosis by neurologists [16] and receiving antiparkinsonism medications. To exclude patients with possible secondary parkinsonism, patients who had ever been diagnosed with dementia, cerebrovascular diseases, head trauma, or psychotic disorders at or one year before the diagnosis of PD were not included in our study. A previous validation study using a hospital administrative database reported a positive predictive value of more than 90% using this definition [17].

### 2.3. Antiparkinsonism Prescribing Analysis

We identified PD with patients who had received outpatient prescriptions for antiparkinsonism drugs including levodopa, carbidopa, bromocriptine mesylate, pergolide mesylate, amantadine, selegiline, anticholinergics, cabergoline, ropinirole, or pramipexole (anatomical therapeutic chemical classification system codes are provided in Supplementary Table 1 in Supplementary Material available online at http://dx.doi.org/10.1155/2016/1859321). Since each patient may have changes of medications throughout the year, our unit of observation was antiparkinsonism prescriptions, which could include more than one antiparkinsonism drug. Each prescription was classified as either monotherapy or combination therapy. Monotherapy was further divided into the levodopa, DA, and other antiparkinsonism agents groups, whereas combination therapy was divided into the levodopa + DA, levodopa + others, DA + others, and levodopa + DA + others groups. The number of PD patients prescribed each monotherapy and each drug category during each period was calculated as a proportion of the number of all PD patients prescribed antiparkinsonism drugs in that period. For patients prescribed ergot DAs before 2007, the year that there were regulatory actions for ergot DAs in April 2007, we confirmed whether these drugs were continued after 2008 or the patients switched to nonergot DAs thereafter. The number of ergot agent users who switched to nonergot DAs was examined.

### 2.4. Statistical Analysis

We calculated the proportion of prescriptions belonging to each particular antiparkinsonism therapy category, whether it was a monotherapy or a combination therapy, during each year to examine the time trend for use of antiparkinsonism agents in Taiwan. The mean number of prescriptions for PD patients was also calculated using the estimated total number of PD patients in Taiwan. We also performed stratified analyses to examine prescription pattern changes for antiparkinsonism drugs in the different age groups of patients with PD. Patients were classified according to age (<60 or ≥60 years). Calendar year, as the continuous variable, was used in the model for linear trends. For results indicating significant trends in each year, the percent change in incidence and prevalence at the end of the study period (2011) was compared to that at the beginning of the study (2004). A two-sided *p* < 0.05 was considered significant. All statistical analyses were performed with SAS 9.2 (SAS Institute, Cary, NC).

## 3. Results

**Figure 1:**
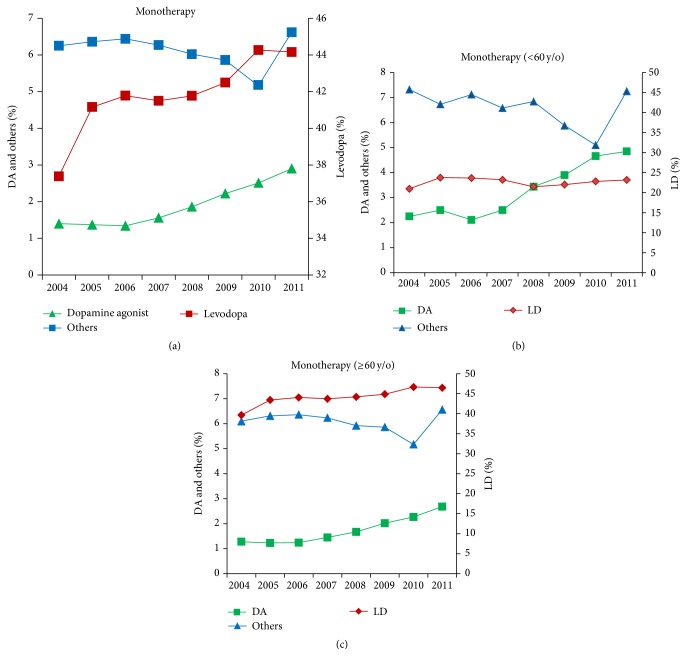
Time trends for antiparkinsonism monotherapy prescription patterns from 2004 to 2011 for the total population (a) stratified by age <60 years (b) or ≥60 years (c). LD: levodopa; DA: dopamine agonist.

A total of 19,302 patients in 2004 and 41,606 patients in 2011 fulfilling the study criteria for PD were included in the analysis. The average age-standardized prevalence of PD per 100,000 was 84.8 in 2004 and 147.7 in 2011, accounting for a 7.9% increase each year [2]. The total number of antiparkinsonism prescriptions increased significantly from 187,137 in 2004 to 414,587 in 2011 (Table 1). Levodopa was the mainstay as monotherapy or in combination with other antiparkinsonism medications. Levodopa monotherapy comprised approximately 37.4% of prescriptions in 2004, increasing slightly to 44.2% in 2011, with an 18.14% increase over the 8-year follow-up period (Table 1 and Figure 1(a)). Combination therapy including levodopa with either DA or other antiparkinsonism medications was 54.45% in 2004 and decreased to 45.08% in 2011. DA monotherapy comprised 1.4% of prescriptions in 2004 and then significantly increased to 2.9% in 2013 (*p* = 0.0004), with an increase rate of 107.15% over the 8-year follow-up period (Table 1 and Figure 1(a)). The percentage of other antiparkinsonism prescriptions remained similar over the 8-year period (6.25% in 2004 and 6.62 in 2011, *p* = 0.40). Notably, as stratified by the two age groups (<60 versus ≥60 years), the prescription patterns differed significantly (Table 2). Although levodopa monotherapy remained the most commonly prescribed medication in the younger-aged PD group, the proportion of prescriptions decreased significantly compared to the older-aged group (about 20% versus 40–46% in the group aged ≥60 years, Figures 1(b) and 1(c)) and the trend was steady over the 8-year study period (20.96% in 2004 and 23.17% in 2011, *p* = 0.79, Figure 1(b)). On the contrary, DA monotherapy comprised 2.25% of prescriptions in 2004 and then increased to 4.85% in 2011 in younger-aged patients (*p* = 0.0006, Figure 1(b)), and the percentage of DA monotherapy prescribed was almost twofold higher compared to the older-aged group (Figure 1(c)).

**Figure 2:**
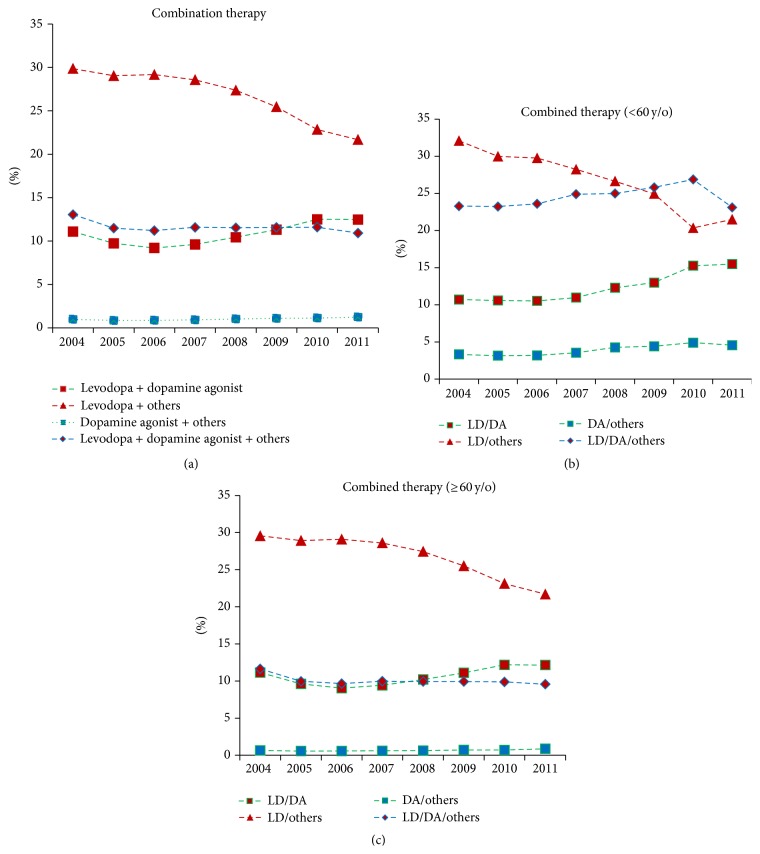
Time trends for antiparkinsonism combination therapy prescription patterns from 2004 to 2011 for the total population (a) stratified by age <60 years (b) or ≥60 years (c). LD: levodopa; DA: dopamine agonist.

Among the combination therapies, levodopa + other antiparkinsonism medications (other than DA) was the most commonly prescribed regimen and comprised 29.86% of prescriptions in 2004 and then decreased to 21.68% in 2011 (*p* = 0.0004, Table 1 and Figure 2(a)). However, combinations of levodopa + DA and DA + other antiparkinsonism medications increased significantly over the 8-year period (Table 1). Of patients receiving combination therapy, 11.09% were prescribed levodopa + DA in 2004, which increased to 12.48% in 2011 (*p* = 0.05). DA + other antiparkinsonism medications comprised 0.98% of prescriptions in 2004 and then increased to 1.24% in 2011 (*p* = 0.006). When stratified by age group, the percentage prescribed DA + other antiparkinsonism medications in the younger-aged group increased almost fourfold compared to the older-aged group (around 3.5–4.6% versus 0.65–0.87% in the older-aged group) (Table 2 and Figures 2(b) and 2(c)). Notably, DA combined with levodopa and other antiparkinsonism medications became the major treatment regimen for younger-aged patients after 2009 (23–25% versus 9-10% in the older-aged patient group, Table 2 and Figure 2(b)).

**Figure 3:**
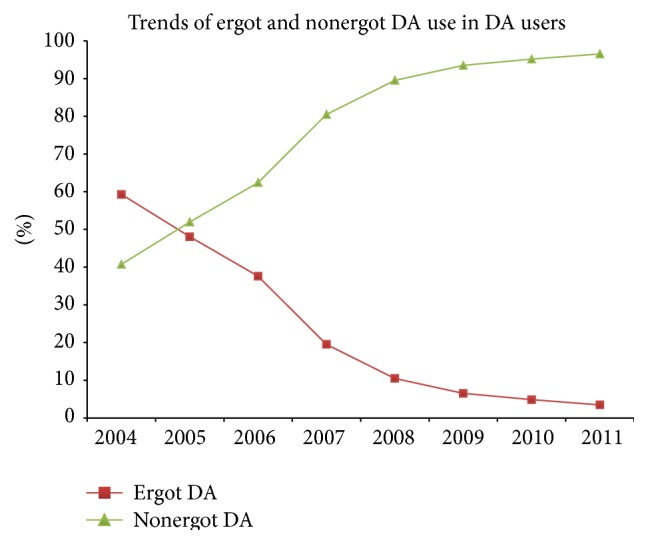
Time trends for ergot and nonergot DA prescriptions among DA users from 2004 to 2011. DA: dopamine agonist.

These findings suggest that there was an increasing trend towards prescribing DA as monotherapy or combination therapy for PD treatment over the 8-year study period in Taiwan. When further examining the classes of DAs, prescriptions of ergot DA dramatically decreased over the time period, especially after 2005 (Figure 3). Nonergot DA prescriptions increased substantially after 2005 and comprised more than 90% of DA agents after 2009. Since the regulatory advisories of use of ergot DA were issued in 2007, we analyzed the number of ergot DA users who switched to nonergot DA in this time period. We observed that the number of patients who switched from ergot to nonergot DA significantly increased after 2007 (*p* = 0.04) and number of ergot users steeply decreased at the same time period (*p* < 0.0001, Supplementary Table 2).

## 4. Discussion

In this large-cohort study assessing time trends for antiparkinsonism prescription patterns in an Asian PD population, we observed that levodopa as monotherapy or in combination with other antiparkinsonism medications was the mainstay therapy for PD patients. Although the use of levodopa monotherapy increased during this 8-year follow-up period, with a total increase of 18.14%, the use of DA increased more than twofold from 2004 to 2011, especially in younger-aged patients with PD. Among DAs, nonergot DAs comprised more than 90% of prescriptions after 2008. For combination therapies, levodopa combined with other antiparkinsonism drugs or DA remains the main therapy regimen for treating PD. However, the combination of DA and other antiparkinsonism therapies became the main treatment regimen for younger-aged patients with PD after 2009. After 2005, the proportion of ergot DA usage markedly decreased and PD patients using nonergot DA increased.

Our findings showing an increase in the use of antiparkinsonian agents, including levodopa and DA, over an 8-year time period have also been reported in other countries [13, 14, 18, 19]. When comparing the changes in levodopa use with those of other countries over similar time periods, the magnitude of increase in levodopa use was similar to that described in Japan from 2005 to 2011, in New Zealand during the 17-year period from 1995 to 2011, and in one community in Spain during a 12-year period from 1992 to 2004 [12–14]. The possible contributors to this increase include an increased prevalence of patients with PD and an increasing number of people being treated for PD. Our group has previously shown that the average age-standardized prevalence of PD per 100,000 was 84.8 in 2004 and 147.7 in 2011 in Taiwan, with an annual increase rate of 7.9% [2]. Another possible reason is the increased lifespan and size of the aging population in recent decades with a resultant increase in the duration of PD treatment per patient. Taiwan has become an aging society since 1993 when the percentage of the population aged older than 65 years reached 7% and increased rapidly to 10.2% by the end of 2007 [20]. In addition, a change in PD treatment strategy may have also influenced the increasing use of levodopa in recent years. The results of the Earlier versus Later Levodopa Therapy in Parkinson's Disease (ELLDOPA) suggested that levodopa may slow the progression of PD [21]. In the ELLDOPA study, 16.5% of patients receiving 600 mg/day levodopa developed dyskinesia in 2 years compared with 2-3% in patients receiving 300 mg/day [21]. The recently published results of the PD MED study, a randomized prospective trial comparing initial treatments of levodopa and levodopa-sparing agents (including DA and MAOBI), also support the beneficial role of levodopa in PD treatment [22]. In the PD MED study, patients treated with levodopa had a higher quality of life and only a slightly increased rate of dyskinesia (36% in the levodopa group versus 33% in the levodopa-sparing group) and no difference in motor fluctuation after 7 years of follow-up, which included 1620 patients with early PD [22]. Multiple large clinical trials have demonstrated that levodopa provides the greatest symptomatic benefit for PD and is associated with less freezing, somnolence, edema, hallucinations, and risk of impulse control disorders than DA [23]. Therefore, there is a trend towards treating patients with PD with levodopa early rather than waiting until the motor symptoms become prominent.

Our study also showed a steeply increased trend towards DA use from 2004 to 2011 as monotherapy or in combination therapy with levodopa or other antiparkinsonism medications. This trend was most significant in the younger-aged patient group. Although it is widely appreciated and accepted that levodopa is the most effective treatment for the motor symptoms caused by PD [7], there is still concern that long-term use of levodopa increases the risk of motor complications, especially dyskinesia, compared with DA [4, 5, 22]. Since younger age-of-onset of PD is a risk factor for dyskinesia [24], DA is usually introduced as the initial treatment for patients aged younger than 60 years [7]. Consistent with the suggested PD treatment guidelines, our results show a substantially increased rate of DA prescribing after 2007, as either monotherapy or combination therapy, in patients aged less than 60 years. However, our results conflicted with one previous study examining the initial prescription patterns in newly diagnosed PD patients in Taiwan from 2000 to 2010, which claimed that only 4.2% of PD patients were prescribed DA [25]. One of the reasons that contribute to the result differences is the definition of PD patient enrollment. We enrolled PD patients that were diagnosed by neurologists, while Guo et al. enrolled PD patients according to ICD 9 diagnostic codes not limited to neurologists, which may result in the findings that DA monotherapy only contributed to 3-4% of their PD patients [25]. Our results are also contrary to those from the New Zealand study, which showed a decreased trend towards DA prescribing, with pergolide showing an increased prescribing trend in their study [12]. Among the reasons for these differences may be that the New Zealand study did not enroll cohorts prescribed one of the nonergot DAs, pramipexole, and also could reflect variability in the availability of individual drugs or differences in clinical practice.

Among DAs, since ergot DA related cardiac valvulopathy was first noticed in 2003 [8, 9], there was a safety concern in using ergot DA and there was a tendency to shift from ergot DA to nonergot DA, which poses a lower risk of cardiac valvulopathy [26]. Nonergot DA was first launched in Taiwan in 2004. Since then, the proportion of nonergot DA prescriptions has increased rapidly and quickly surpassed ergot DA by the next year. The substantially increasing trend toward nonergot DA use is comparable with a Japanese study in which nonergot DA comprised 17.9% of prescriptions in 2005 and then surpassed ergot DA in 2008 [14]. However, recent studies with long-term follow-up of patients participating in levodopa versus DA clinical trials have shown remarkably similar outcomes with respect to both parkinsonian features and motor complications independent of how treatment was initiated [6, 22]. Since nonergot DAs cost more compared to levodopa, the cost-effectiveness benefit is another important factor for physicians to weigh when judging which treatment to initiate for younger-aged patients in the future.

Our study provides a descriptive overview of how the use of antiparkinsonism agents has changed over the past 8 years in Taiwan. We analyzed trends for use of both monotherapy and combination therapies in PD patients and stratified the results by age group, providing a more accurate evaluation of antiparkinsonism prescribing trends. Our study has the following strengths. First, we used nationwide data and had a large sample size, which depicts unbiased temporal trends for antiparkinsonism prescription patterns in our population. Second, the NHI program in Taiwan provides continuing universal coverage for the entire population of Taiwan, which avoids selection bias. Third, the NHI datasets were used, which eliminated the need to minimize the number of patients in the cohorts lost to follow-up. Finally, a large sample of geographically dispersed patients was easily obtained, avoiding regional differences in estimation. However, our study also has some limitations. First, our diagnosis of PD was based on the diagnostic code from the NHI database; therefore, we could not distinguish between primary and secondary parkinsonism. However, we excluded patients who had ever had diagnoses of dementia, cerebrovascular diseases, head trauma, or psychotic disorders at the time of or one year before the diagnosis of PD to exclude the possibility of enrolling patients with diffuse Lewy body disease, vascular parkinsonism, and secondary parkinsonism related to intracranial hemorrhage or neuroleptic use. We were unable to review the medical records of all defined patients with PD since all the medical information from the national NHI database was deidentified because of ethical requirements; therefore, we have no clinical data such as neuroimaging examinations or the duration of and treatment response to antiparkinsonism medications in the defined patients with PD.

In conclusion, our results show that levodopa, either as monotherapy or combination therapy, is the mainstay treatment for patients with PD in Taiwan from 2004 to 2011. During this 8-year time period, there was a decrease in prescriptions for ergot DAs and an increase in prescriptions for nonergot DAs after the regulatory actions occurring in 2007. Neurologists have different treatment strategies for different age groups of patients with PD, and DA, especially nonergot DA, was predominantly prescribed in younger-aged PD patients. Further studies are needed to evaluate whether this treatment pattern will lead to improved clinical outcomes in a cost-effective way.

## Supplementary Material

The anatomical therapeutic chemical classification system codes of anti-parkinsonism medications analyzed in the current study are provided in Supplementary Table 1.

## Figures and Tables

**Table 1:** Antiparkinsonism drug prescription patterns in Taiwan from 2004 to 2011.

	2004	2005	2006	2007	2008	2009	2010	2011	*p* for trend
Number of prescriptions	187,137	239,243	282,270	321,108	355,219	383,173	412,751	414,587	<0.0001
Monotherapy									
Levodopa^a^	37.38	41.16	41.78	41.5	41.77	42.49	44.27	44.16	0.003
Dopamine agonist	1.40	1.37	1.34	1.56	1.86	2.22	2.51	2.90	0.0004
Others^b^	6.25	6.36	6.44	6.27	6.02	5.86	5.16	6.62	0.40
Combination therapy									
Levodopa^a^ + dopamine agonist	11.09	9.74	9.20	9.60	10.44	11.30	12.50	12.48	0.05
Levodopa^a^ + others^b^	29.86	29.03	29.17	28.56	27.37	25.45	22.84	21.68	0.0004
Dopamine agonist + others^b^	0.98	0.86	0.87	0.92	1.02	1.10	1.13	1.24	0.006
Levodopa^a^ + dopamine agonist + others^b^	13.05	11.48	11.20	11.58	11.53	11.57	11.59	10.92	0.10

^a^Levodopa alone and combination of levodopa and dopa-decarboxylase inhibitors.

^b^Others include amantadine, selegiline, rasagiline, and entacapone.

**Table 2:** Antiparkinsonism drug prescription patterns by age groups (<60 versus ≥60 years) in Taiwan from 2004 to 2011.

	2004	2005	2006	2007	2008	2009	2010	2011	*p* for trend
Age < 60 years									
Number of prescriptions	22,628	27,395	31,167	34,783	37,876	39,810	41,431	41,225	<0.0001
Monotherapy									
Levodopa^a^	20.96	23.76	23.64	23.22	21.48	22.00	22.81	23.17	0.79
Dopamine agonist	2.25	2.50	2.11	2.50	3.44	3.90	4.66	4.85	0.0006
Others^b^	7.32	6.73	7.12	6.58	6.84	5.88	5.10	7.25	0.25
Combination therapy									
Levodopa^a^ + dopamine agonist	10.73	10.59	10.54	10.99	12.31	13.01	15.27	15.50	0.0009
Levodopa^a^ + others^b^	32.10	29.99	29.77	28.25	26.65	24.95	20.36	21.51	<0.0001
Dopamine agonist + others^b^	3.34	3.17	3.21	3.56	4.28	4.43	4.92	4.58	0.001
Levodopa^a^ + dopamine agonist + others^b^	23.30	23.25	23.61	24.91	25.00	25.83	26.89	23.13	0.21
Age ≥ 60 years									
Number of prescriptions	164,509	211,848	251,103	286,325	317,343	343,363	371,320	373,362	<0.0001
Monotherapy									
Levodopa^a^	39.64	43.41	44.04	43.72	44.19	44.87	46.66	46.48	0.003
Dopamine agonist	1.28	1.23	1.24	1.45	1.67	2.02	2.27	2.68	0.0004
Others^b^	6.10	6.31	6.36	6.23	5.92	5.86	5.17	6.56	0.48
Combination therapy									
Levodopa^a^ + dopamine agonist	11.14	9.62	9.04	9.43	10.21	11.10	12.19	12.15	0.09
Levodopa^a^ + others^b^	29.55	28.91	29.10	28.60	27.45	25.51	23.12	21.70	0.0006
Dopamine agonist + others^b^	0.65	0.56	0.58	0.60	0.63	0.71	0.70	0.87	0.02
Levodopa^a^ + dopamine agonist + others^b^	11.64	9.96	9.66	9.97	9.92	9.92	9.88	9.57	0.09

^a^Levodopa alone and combination of levodopa and dopa-decarboxylase inhibitor.

^b^Others include amantadine, selegiline, rasagiline, and entacapone.
