# Supplementary material for: National Trends of Antiparkinsonism Treatment in Taiwan: 2004–2011
Source: Parkinsons Dis. 2016 Feb 18;2016:1859321. doi: 10.1155/2016/1859321 (PMC4775810; doi:10.1155/2016/1859321)
Supplement: Supplementary file 1 — The anatomical therapeutic chemical classification system codes of anti-parkinsonism medications analyzed in the current study are provided in Supplementary Table 1. [file 1859321.f1.docx]

Supplementary Table 1. Anatomical therapeutic chemical classification system (ATC) codes for anti-Parkinsonism medications

| **Type** | **Drugs** | **ATC code** |
| --- | --- | --- |
| **L-dopa** | Levodopa | N04BA01 |
|  | Levodopa and decarboxylase inhibitor | N04BA02 |
|  | Levodopa, decarboxylase inhibitor and COMT inhibitor | N04BA03 |
| **Ergot DA** | Bromocriptine | N04BC01 |
|  | Pergolide | N04BC02 |
|  | Cabergoline | N04BC06 |
| **Non-ergot DA** | Ropinirole | N04BC04 |
|  | Pramipexole | N04BC05 |
|  | Rotigotine | N04BC09 |
| **Others** | Amantadine | N04BB01 |
|  | Monoamine oxidase inhibitors  Selegiline  Rasagiline | N04BD01  N04BD02 |
|  | Entacapone | N04BX02 |

DA: dopamine agonist

Supplementary Table 2. Number of PD patients who switched from ergot-DA to non-ergot DA during 2004-2011.

|  | 2004 | 2005 | 2006 | 2007 | 2008 | 2009 | 2010 | 2011 | *p* for trend |
| --- | --- | --- | --- | --- | --- | --- | --- | --- | --- |
| Number of patients | 19,302 | 23,875 | 27,604 | 31,176 | 33,904 | 36,618 | 39,256 | 41,606 | <0.0001 |
| Number of patients received ergot DA | 3,731 | 3,270 | 2,759 | 2,025 | 1,062 | 746 | 664 | 488 | <0.0001 |
| Number of patients switched from ergot to non-ergot DA | 336 | 327 | 359 | 770 | 212 | 104 | 126 | 83 | 0.04 |
